# Strategies for the Preservation, Restoration and Modulation of the Human Milk Microbiota. Implications for Human Milk Banks and Neonatal Intensive Care Units

**DOI:** 10.3389/fmicb.2018.02676

**Published:** 2018-11-09

**Authors:** Leónides Fernández, Lorena Ruiz, Josué Jara, Belén Orgaz, Juan M. Rodríguez

**Affiliations:** ^1^Department of Galenic Pharmacy and Food Technology, Complutense University of Madrid, Madrid, Spain; ^2^Department of Microbiology and Biochemistry of Dairy Products, Instituto de Productos Lácteos de Asturias – Consejo Superior de Investigaciones Científicas, Villaviciosa, Spain; ^3^Department of Nutrition and Food Science, Complutense University of Madrid, Madrid, Spain

**Keywords:** human milk, microbiota, probiotics, lactic acid bacteria, minimal microbiota, preterm infants, NICU, human milk bank

## Abstract

Studies carried in the last years have revealed that human milk contains a site-specific microbiota and constitutes a source of potentially beneficial bacteria to the infant gut. Once in the infant gut, these bacteria contribute to the assembly of a physiological gut microbiota and may play several functions, contributing to infant metabolism, protection against infections, immunomodulation or neuromodulation. Many preterm neonates are fed with pasteurized donor’s human milk (DHM) or formula and, therefore, are devoid of contact with human milk microbes. As a consequence, new strategies are required to allow the exposition of a higher number of preterm infants to the human milk microbiota early in life. The first strategy would be to promote and to increase the use of own mother’s milk (OMM) in Neonatal Intensive Care Units (NICUs). Even small quantities of OMM can be very valuable since they would be added to DHM in order to microbiologically “customize” it. When OMM is not available, a better screening of donor women, including routine cytomegalovirus (CMV) screening of milk, may help to avoid the pasteurization of the milk provided by, at least, a relevant proportion of donors. Finally, when pasteurized DHM or formula are the only feeding option, their supplementation with probiotic bacteria isolated from human milk, such as lactic acid bacteria or bifidobacteria, may be an alternative to try to restore a human milk-like microbiota before feeding the babies. In the future, the design of human milk bacterial consortia (minimal human milk microbiotas), including well characterized strains representative of a healthy human milk microbiota, may be an attractive strategy to provide a complex mix of strains specifically tailored to this target population.

## From Own Mother’s Milk to Donor Human Milk: The “Fall” of the Human Milk Microbiota

Fresh human milk is considered as the gold standard of infant nutrition because it provides all the nutrients and vitamins required for the optimal development of the infant (American Academy of Pediatrics [AAP], 2012). In addition, it contains an inimitable plethora of bioactive factors, including soluble immune factors, antimicrobial proteins and peptides, functional fatty acids, hormones, oligosaccharides, stem cells, and microbes. This vast array of bioactive compounds act synergistically in order to preserve infants’ health, making difficult to delineate the specific functions of a given milk component without taking in account its potential interactions with other human milk ingredients. This fact reflects the effect of evolution selection toward a perfect multifunctional species-specific fluid that meets the complex infant requirements during early life (Hennet and Borsig, 2016).

The consideration of human milk as a gold standard for term and preterm infant feeding usually refers to own mother’s milk (OMM) since its composition is adapted to the specific needs of each particular infant (gestational age, time of the day, geographical location, environment, etc,) which converts this biological fluid into the ultimate personalized food and medicine (Furman et al., 2003; Meinzen-Derr et al., 2009; Corpeleijn et al., 2012; Chowning et al., 2016; Hennet and Borsig, 2016). Donor human milk (DHM) is the best alternative when there is no OMM available or there is no enough OMM to guarantee a proper nutrition of the preterm infant (American Academy of Pediatrics [AAP], 2012; ESPGHAN Committee on Nutrition et al., 2013; Quigley and McGuire, 2014; DiLauro et al., 2016; Sisk et al., 2017).

The microbiological composition of DHM is a relevant concern for Human Milk Banks (HMBs) since it will be ingested mainly by preterm or sick term infants who are known to be particularly susceptible to infections. Under physiological conditions, the bacterial concentration of human milk is usually low (<3 log cfu/ml) and is dominated by Gram-positive bacteria (staphylococci, streptococci, corynebacteria, propionibacteria, lactic acid bacteria and bifidobacteria, among others), which are the core components of the human milk microbiota (Fernández et al., 2013). However, milk extraction devices and the subsequent management of the collected milk may lead to the bacterial contamination of DHM. For instance, the use of milk pumps may result in a high concentration (even higher than 6 log cfu/ml) of contaminating Gram-negative bacteria (enterobacteria, *Pseudomonas*, *Stenotrophomonas*, etc.) and yeasts arising from rinsing water and/or poor hygienic manipulation practices (Boo et al., 2001; Brown et al., 2005; Cervia et al., 2008; Landers and Updegrove, 2010; Fihman et al., 2012; Decousser et al., 2013; Cohen et al., 2017; Jiménez et al., 2017). Such deleterious microorganisms may adhere to pumps’ surfaces and resist usual cleaning and disinfection procedures (Marín et al., 2009). Contaminated human milk has occasionally been associated to infections of preterm infants by microorganisms such as *Escherichia coli*, *Pseudomonas* sp., *Klebsiella* sp., or *Serratia* sp. (Lanzieri et al., 2013; Kayıran et al., 2014; Nakamura et al., 2016). In addition, high-risk viruses that can be potentially transferred from mother to infant through breastfeeding, such as human immunodeficiency viruses (HIV-1 HIV-2), cytomegalovirus, papillomaviruses, human T-lymphotrophic viruses (HTLV-I, HTLV-II), as well as Ebola, Marburg or Zika viruses, are of special concern (Diaz et al., 2018; Mann et al., 2018).

Globally, it means that, on one hand, donors have to receive a thoughtful formation on hygienic practices while collecting and preserving their milk; and, on the other hand, that a proper microbiological screening of donor women must be carried out in HMBs. Although some criteria may differ among the HMBs of different countries, this usually includes the fulfillment of a questionnaire regarding lifestyle, diseases and risk factors, the serologic screening of donors, and the pre- and/or post-pasteurization microbiological analysis of the milk (Arslanoglu et al., 2010; NICE, 2010). The exclusion criteria for donor women generally include the following: (a) having (she or her partner) positive blood results for hepatitis B, hepatitis C, HTLV-I, HTLV-II, HIV-1, HIV-2, or syphilis; (b) suffering from other sexually acquired infections (human papillomavirus, ano-genital infection by herpes simplex virus types 1 and 2, infections by *Chlamydia trachomatis*, *Neisseria gonorrheae*, or *Trichomonas vaginalis*) since it may imply a high risk sexual behavior (and, due to the same reason, women with a promiscuous sexual life are also excluded); (c) having a disease that requires frequent blood (or blood derivatives) transfusions; and (d) suffering or having a family history of spongiform transmissible encephalopathy.

Despite the donor microbiological screening, DHM is pasteurized in most HMBs to further ensure microbiological safety by killing all non-spore forming microorganisms, including all the high-risk viruses cited above (Orloff et al., 1993; Chiavarini et al., 2011; Rigourd et al., 2011; Donalisio et al., 2014; Hamilton Spence et al., 2017; Pfaender et al., 2017). Alternative emerging non-thermal food technologies are being tested in order to preserve the biological activities of some of the human milk compounds (immunoglobulins, cytokines, enzymes, etc.) but all of them also destroy non-sporulated bacteria. Obviously, pasteurization does not only kill potentially harmful microorganisms but also destroy the beneficial milk microbiota. As stated above, diverse culture-dependent and –independent studies have shown that human milk contains a site-specific microbiota, which is subsequently transferred to the infant (Makino et al., 2015; Milani et al., 2015; Asnicar et al., 2017; Duranti et al., 2017; Murphy et al., 2017; Pannaraj et al., 2017), playing an important role in the acquisition and evolution of the microbiota in early life, and contributing to metabolic functions and to the maturation of the immune and neuroendocrine systems (Fernández et al., 2013; Jeurink et al., 2013; Jost et al., 2015; McGuire and McGuire, 2017).

In the past, it was thought that introduction of solid foods changed the infant gut microbiome toward an adult-like microbiome, independently of whether the infant was receiving human milk or not; however, it has been recently described that infant microbiota is dominated by bacteria provided by human milk as long as they are breastfed, independently of whether solid foods are introduced or not (Bäckhed et al., 2015). In other words, the gut microbiomes of babies who stopped breastfeeding earlier are dominated by adult-like taxa, while those of babies breastfed for longer are dominated by bacteria present in human milk. The conclusion that stopping breastfeeding -rather than introducing solids-drives gut microbiota maturation is an important paradigm’s shift and indicates that the impact of human milk bacteria on infant gut microbiota seems to be much stronger than previously thought.

**FIGURE 1:**
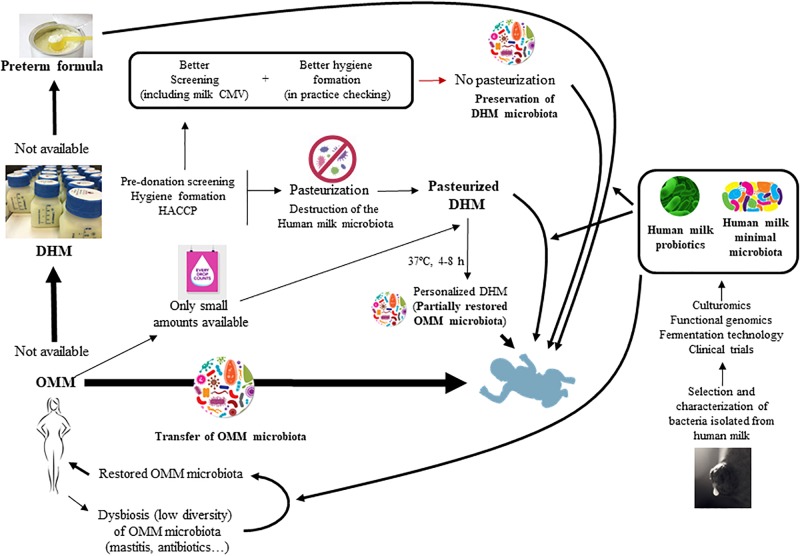
Strategies for the restoration and modulation of the human milk microbiota and its application to preterm infants.

As a consequence, devising new strategies to allow that preterm infants may receive the human milk microbiota might significantly improve the gut microbiota establishment and its associated health benefits in this population (Figure 1). Some of these strategies will be discussed in this review.

## Increasing of the Use of OMM in NICUs: Benefits and Challenges

The best way to increase the number of preterm neonates exposed to their mothers’ milk microbiota is increasing the use of OMM for preterm neonates at NICUs. In the last years, some authors have criticized the fact that the terms “*human milk*” or “*human milk feeding*s” are often used interchangeably for both OMM and DHM (Meier et al., 2017a), as the first provides much more benefits to preterm infants that DHM (Vohr et al., 2006, 2007; Colaizy and Morriss, 2008; Belfort et al., 2016; Meier et al., 2017a). Although in this section the benefits of OMM over DHM will be highlighted, it must be said that DHM is superior to formula in terms of infant development and protection against diseases and their use in NICUs is very valuable to provide some of the biologically active compounds that remain totally or partially active after the heat treatment those preterm infants that have no access to OMM.

DHM differs significantly from OMM in, at least, three main aspects: (a) pasteurization decreases or eliminates the biological activity of many milk bioactive compounds (as discussed below); (b) cold storage and freeze-thaw cycles that are inherent in the storage and processing of DHM can also alter milk composition and functionality; and (c) there may be relevant differences in the stage of lactation for which DHM replaces OMM. Regarding the later point, mature DHM frequently replaces OMM colostrum and transitional milk (Meier et al., 2017a). In this context, it must be highlighted that the concentrations of many milk bioactive compounds decline (in some cases quite rapidly) during lactation, as the infant matures. In addition, feeding preterm infants with DHM obtained from mothers of much older infants has been considered a major gap in lactation and neonatology knowledge (Meier et al., 2017a). DHM needs fortification with protein, energy, vitamins and minerals (Moro et al., 2015), and usually has a lower protein content in relation to milk produced during early lactation (Schanler, 2007; Valentine et al., 2010). The relationship observed between a lower growth rate and impaired neurological development is particularly worrying (Ramel et al., 2012). In this sense, colostrum and milk from mothers of preterm infants seem to specifically potentiate immunomodulatory and nutritional programming as well as selective organ growth, including the immature brain (Isaacs et al., 2010; Sherman et al., 2015). Individualized (adjustable or targeted) fortification of DHM is recommended (Arslanoglu et al., 2009; ESPGHAN Committee on Nutrition et al., 2013; Moro et al., 2015).

Immunologically, human milk is usually suited to the gestational age at which a baby is born (Espinosa-Martos et al., 2013; Kaingade et al., 2017). Colostrum and transient milk from mothers of very preterm infants seems to contain higher amounts of key immune compounds than those from mothers of term infants (Montagne et al., 1999; Araujo et al., 2005; Koenig et al., 2005; Castellote et al., 2011; Espinosa-Martos et al., 2013; Moles et al., 2015b), indicating that the degree of immunoprotection provided through breastfeeding is highly correlated with the immaturity degree of the neonate. These facts further support the high relevance of OMM for preterm infants, whose risk for feeding intolerance, necrotizing enterocolitis and sepsis is higher than that of term infants (Westerbeek et al., 2006).

Diverse OMM components that are thought to benefit preterm health, including neurodevelopmental advantages, are reduced or absent in DHM, and include, among others, immunoglobulins (Contador et al., 2013), some cytokines, chemokines and growth factors (Espinosa-Martos et al., 2013), hormones and enzymes (Peila et al., 2016), myoinositol (de Segura et al., 2012; Espinosa-Martos et al., 2013; Moles et al., 2015b) and soluble CD14 (Cossey et al., 2009). Human milk oligosaccharides (HMOs) are other of the human milk components thought to have numerous beneficial functions in infants, from prebiotics to immunomodulation and protection against infectious diseases. Although our knowledge on the effect of pasteurization on this type of compounds is still limited and controversial, recent data have revealed that infants in NICUs who receive DHM are likely to ingest HMOs at different total amounts and relative composition from what they would receive with their OMM (Marx et al., 2014). Concentrations of HMOs in human milk show variations that depend on the secretor type of the mother and the lactation period (Thurl et al., 2017); therefore, OMM is again the best source of HMOs for a preterm infant. Finally, and in contrast to either formula or DHM, OMM contains an array of mother-specific probiotic and commensal bacteria (the milk microbiota), that may selectively use the highly complex and individual HMOs as prebiotics to promote their own growth once inside the infant gut (Jost et al., 2015). It is probable that exogenous probiotics cannot fully replace OMM ones because the later are specifically suited to the HMOs existing in the milk of the same woman. Pasteurization completely eradicates OMM bacteria, including lactic acid bacteria and bifidobacteria, potentially displacing or altering the impact of OMM microbiota on infant gut colonization.

Many initiatives are useful to increase the rates of OMM feeding when a preterm infant leaves the NICU and most lactation barriers are modifiable when evidence-based practices and resources are prioritized (Meier et al., 2017b). The research literature is replete with strategies to acquire and feed OMM in the NICU. Unfortunately, many of them are not implemented in practice because they require an initial economical investment (Meier et al., 2017b), despite the fact that OMM-based programs are, at the medium and long term, more economical than DHM-oriented ones and provides greater protection from acquired morbidities (Jegier et al., 2013; Patel et al., 2013; Johnson et al., 2015). As an example, and although it may not be the general rule, a study has warned of a decrease in the cumulative proportion of OMM received by very low-birth-weight (VLBW) infants (i.e., with weight <1,500 g).

VLBW infants at 14 and 28 days post-birth after the introduction of DHM into a NICU in which 98% of these infants had received some OMM prior to DHM availability (Meier et al., 2017b). Problems that mothers of preterm neonates have to face in order to provide OMM at the early post-birth period and to keep breastfeeding their infants after NICU discharge have been reviewed recently (Meier et al., 2017a,b). These facts should be a matter of reflection by HMBs and NICU’s managers.

## DHM: to Heat or Not to Heat?

HMBs are the most important providers of DHM to NICUs and there is no doubt that they have played a decisive role in extending human milk benefits to preterm infants andother high-risk infants (American Academy of Pediatrics [AAP], 2012; ESPGHAN Committee on Nutrition et al., 2013; World Health Organization [WHO], 2017). DHM is pasteurized in most HMBs although in a few countries, such as Japan and Norway, there is a history of using raw non-pasteurized DHM to feed preterm infants, a practice that preserves milk bioactive compounds, including the microbiota. This means that use of non-pasteurized DHM is feasible and, with appropriate control, as safe as that of pasteurized DHM (Grøvslien and Grønn, 2009; Mizuno et al., 2015). To ensure its safety, unpasteurized milk may be considered when extremely strict donor screening, including regularly repeated testing, is performed, when there is complete traceability of milk from donor to consumer, and when the HIV and hepatitis rates are low.

Fear to transmission of pathogenic microorganisms [particularly cytomegalovirus (CMV) in Western countries] is the basis of human milk pasteurization; however, it is also true that use of OMM has an extraordinary history of safety despite women that provide OMM to their preterm infants are not screened for CMV and no differences in the level of hygiene formation have been reported between OMM and DHM providers so far. It is somehow shocking, and again a reason for reflection, that with the current arguments given to pasteurize DHM, use of OMM or breastfeeding should be forbidden to many mothers of preterm infants.

It must be also noted that pasteurized DHM is not devoid of microbiological risks; although viruses and contaminant vegetative bacterial cells are killed by standard pasteurization processes, the treatment does not guarantee the destruction of spore-forming bacteria (e.g., *Bacillus cereus*) (Landers and Updegrove, 2010; de Segura et al., 2012; Almutawif et al., 2017); subsequently, such bacteria and potential post-pasteurization contaminants can overgrow since the treatment destroys the natural competitors provided by the milk microbiota and, also, alters some of the bacteriostatic compounds of milk (Ford et al., 1977).

In order to avoid milk pasteurization, donors should receive an excellent education and training on the subject while the HMB staff should (a) monitor that such knowledge is applied in practice; and (b) control environmental contamination during DHM collection, distribution and storage (Becker et al., 2015). But, if donors are submitted to a careful pre-donation screening (hepatitis B and C, HTLV I and II, HIV 1 and 2, syphilis, no toxic habits, no sexual risky behavior, etc.) and receive a thoughtful hygienic formation (as it uses to be the case in most Western HMBs), why do HMBs still pasteurize DHM? The most common answer is because of the potential threat of CMV transfer to low birth weight and/or preterm infants. In these populations the risk of CMV transmission through human milk from seropositive mothers has traditionally been considered higher than for term infants (Jim et al., 2009). Freezing of fresh human milk reduces, but does not eradicate infectivity (Lombardi et al., 2012) and does not affect the rate of CMV transmission (Omarsdottir et al., 2015).

In full-term infants, CMV infections are most often asymptomatic because of the transfer of specific maternal protection during pregnancy. On the contrary, preterm infants (and, particularly the VLBW ones) can suffer a symptomatic infection. In spite of this, breastfeeding is usually recommended on a risk-benefit basis since CMV infection resolves spontaneously and without sequelae in most instances (Lombardi et al., 2012; Romero-Gómez et al., 2015). Indeed, several studies found that exposure of preterm infants to CMV-positive human milk did not negatively affect either neurological development or hearing (Yasuda et al., 2003; Vollmer et al., 2004; Jim et al., 2015). Most recently, Gunkel et al. (2018) showed in a cohort study, that postnatal CMV infection in preterm infants had no impact on neurodevelopment or hearing for the first 6 years after birth.

Even in the case that a risk as close to zero has to be assured, routine CMV screening of the milk of donor women may allow the selection of those whose CMV-free milk does not need to be pasteurized. Techniques for a proper diagnose are currently available (Hartleif et al., 2016) and should be implemented at HMBs or at the services of Microbiology of those hospitals harboring a HMB. Knowledge on the rates of CMV-positive lactating women are very scarce or non-existing in many countries. Such data may be useful for HMBs.

## Personalization of Pasteurized Donor Milk With OMM

As stated above, human milk naturally contains microbes, including lactic acid bacteria and bifidobacteria, that constitute a truly site-specific microbiota. Interestingly, each woman seems to contain a unique fingerprint-like milk microbiota that is stable over time (Hunt et al., 2011). Most components of the milk microbiota are transferred to the infant (Martín et al., 2003, 2012; Jost et al., 2013), together with mother-specific HMOs that are specifically suited to her own milk’s microbiota, working synergistically to provide health benefits to the infant (Jost et al., 2015).

The early post-birth lactation stages may be particularly problematic for mothers of preterm infants, who usually depend on pumping for milk expression (Meier et al., 2016). In addition, preterm birth disrupts physiological processes in the mammary epithelium (Cregan et al., 2002; Pang and Hartmann, 2007; Neville, 2009). Under such circumstances, they may be unable to produce enough milk to fully support the nutritional requirements of their babies (Smith et al., 2003; Lee and Gould, 2009). However, such low amounts can be very valuable for microbial personalization of DHM, providing lasting health benefits and encouraging the NICU mothers to continue pumping, as it will be shown below.

In a recent study, pasteurized DHMs were inoculated with small proportions of OMM (ranging from 1 to 30% v:v) and the mixtures were incubated for 4 or 8 h at 37°C (Cacho et al., 2017). The authors showed that it was possible, at least partly, to reestablish the levels of staphylococci, streptococci and lactic acid bacteria to those that were naturally found in the OMM samples, and suggested that the optimal restoration strategy would be adding 10% OMM to DHM and incubating for 4 h. This study also confirmed that the microbiota of human milk has a high degree of inter-individual variability.

This highly individualized method to provide human milk bacteria to preterm infants confers an active role to the mother of a very preterm infant, encouraging milk extraction and the maintenance of lactation in the future. In contrast, some questions and current practical limitations may arise in relation to this approach: (a) is it feasible in the daily management of a NICU? And, more importantly, (b) are ethical committees ready to accept an increase in the milk staphylococcal and streptococcal count as something desirable for a preterm neonate? At the short time the answers seem to be “no” but in the context of the “microbiome era” where fecal transplant has become a kind of standard treatment for pseudomembranous colitis caused by *Clostridium difficile* (see below), it may be a practical and economical approach in a close future. For this purpose, studies including large cohorts and a careful safety and efficacy assessment will be required.

## Preterm Gut Colonization and Probiotics

Premature neonates show numerous signs of organ immaturity responsible for their inability to face postnatal life challenges appropriately. Immediately after preterm birth, the immature gut receives a plethora of microbiological, immunological, and nutritional-related challenges difficult to cope with due to deficiencies in this organ (Siggers et al., 2011; Ruiz et al., 2016), with deep consequences at systemic level (Claus et al., 2008; Neuman et al., 2015).

The gut colonization pattern of in premature infants has been described as delayed and aberrant (Koenig et al., 2011; LaTuga et al., 2011; Arboleya et al., 2012a). This may have important and long lasting health implications since the early neonatal period is a key period for reaching a microbiota-induced host-homeostasis (Hansen et al., 2012; Olszak et al., 2012; Cox et al., 2014). Microbiota provides stimuli necessary for an adequate developmental programming of several metabolic, immunological and neuroendocrine functions, not only in the gastrointestinal tract but in most, if not all, organs (Ley et al., 2008; Renz et al., 2012; Sommer and Bäckhed, 2013). The existence of specific alterations on the microbiota establishment process in preterm infants may contribute to the pathogenesis of different prematurity-related diseases (Moles et al., 2013; Ruiz et al., 2016). Thus, there is an increasing interest in the management of the microbial colonization process in the preterm infant. Among the strategies that can be used with this objective, the administration of selected probiotic bacteria, with appropriate microbiota modulation capabilities, constitutes a promising approach (Arboleya et al., 2012b; Alfaleh and Anabrees, 2014; Ruiz et al., 2016).

Probiotics are defined as “*live micro-organisms which, when administered in adequate amounts, confer a health benefit on the host*” (FAO/WHO, 2002; Hill et al., 2014). Probiotic effects are often strain-specific and the beneficial effects of a given strain for a specific target cannot be extrapolated to other targets. In addition, probiotic effects also depend on posology.

The demand of probiotics has experienced a rapid growth in recent decades, even for administration to high-risk populations, as is the case of premature infants. The initial rationale for administering probiotics to preterm infants was the key role played by the initial colonization of the neonatal intestine in necrotizing enterocolitis (NEC) and sepsis outcomes. The potential benefits of probiotic therapy in this population include improving the intestinal barrier, increasing the production of IgAs and anti-inflammatory cytokines, increasing the diversity and functionality of the microbiota, and decreasing the pathological bacterial translocation (Versalovic, 2013).

The results of routine use in clinical practice, trials, reviews, and meta-analyses confirm that the administration of probiotics to low or VLBW premature infants is safe and significantly reduces the risk of NEC, the mortality due to any cause and the time required for enteral feeding (Alfaleh et al., 2010, 2011; Deshpande et al., 2011; Wang et al., 2012; Bonsante et al., 2013; Srinivasjois et al., 2013; Versalovic, 2013; Athalye-Jape et al., 2014; Johnson-Henry et al., 2016). Nevertheless, the heterogeneity in strains, dose, form of application and duration of treatment, make it difficult or impossible to reach consensus about the most appropriate strains and clear therapy guidelines (Neu, 2011; Ofek Shlomai et al., 2014).

There is an urgent need of identifying the best suited strains for this particular population since, with a few exceptions (Arboleya et al., 2013; Moles et al., 2015a), most of the strains used in preterms had not been selected for this target population so far. Therefore, the identification of the best suited strains and conditions is still a pending task (Ruiz et al., 2016). Taking into account the special vulnerability of this population, a careful evaluation of the potential risks associated with probiotic administration and microbiota modulation in preterm infants is required, including those involving the production and manipulation of these products (Bertelli et al., 2015; Zbinden et al., 2015). In 2014, a probiótico product (ABC Dophilus) used in a preterm clinical trial was recalled by the manufacturer since the Center for Disease Control (CDC) reported a case of mortality due to contamination with the fungus *Rhizopus oryzae* (Food and Drug Administration [FDA], 2014). Despite such events are very scarce, it highlights the need to produce probiotics using pharmaceutical standards (Johnson-Henry et al., 2016).

## Human Milk: A Source of Probiotic Bacteria

Human milk guarantees a constant supply of bacteria (5–7 log bacteria per day) throughout the lactation period (Heikkilä and Saris, 2003; Martín et al., 2003). Among isolated bacteria, lactobacilli and bifidobacteria isolates have received a particular attention because of their long history of safety use and the potential of many strains from these groups to be used as probiotics. Consequently, the isolation of potentially beneficial bacteria from this biological fluid is particularly attractive for pharmacological and nutrition companies since, by their very own nature, they respond to many of the criteria generally required for human probiotics (Fernández et al., 2013; Jeurink et al., 2013).

Among the lactobacilli and bifidobacteria species isolated from human milk, many of them (*Lactobacillus salivarius*, *L. gasseri*, *L. reuteri*, *L. plantarum*, *L. rhamnosus*, *L. fermentum*, *Bifidobacterium breve*, *B. Longum*, or *B. bifidum*), are included among the potentially probiotic ones and enjoy the GRAS (Generally Recognized As Safe; FDA, United States) and the QPS (Qualified Presumption of Safety; EFSA, EU) status (EFSA, 2013). In fact, the probiotic potential of some lactobacilli strains from this biological fluid is similar or higher to that of certain strains commercially available worldwide (Martín et al., 2005, 2006). In general, lactic bacteria and bifidobacteria isolated from human milk appear to play different beneficial roles for the mother-infant dyad (Fernández et al., 2013; Jost et al., 2015). The safety and functionality (enhancement of the intestinal barrier function, antimicrobial activity, protection against infectious diseases, metabolic roles, immunomodulation, and neuromodulation) of some of human milk-derived strains (*L. fermentum* CECT5716, *L. salivarius* CECT5713, *L. salivarius* PS2, *L. gasseri* CECT5714, *L. salivarius*PS12934, and *B. breve*PS12929) have been evaluated *in vitro*, *in silico*, and *in vivo*, both in animal models and in human clinical trials, including pregnant and lactating women, term infants and extremely low birth weight premature infants (Arroyo et al., 2010; Maldonado et al., 2010, 2012; Martín et al., 2010; Gil-Campos et al., 2012; Fernández et al., 2014, 2016; Maldonado-Lobón et al., 2015b; Moles et al., 2015a).

Apart from lactic acid bacteria and bifidobacteria (the classic prototype of probiotic bacteria), other milk bacteria with less glamorous and appealing names, may provide relevant beneficial effects to the infants. Streptococci (mitis and salivarius groups) and coagulase-negative staphylococci (CNS) are among the dominant bacteria both in human milk (Jiménez et al., 2008b; Hunt et al., 2012; Martín et al., 2012; Cacho et al., 2017) and in the feces of breast-fed infants (Lundequist et al., 1985; Sakata et al., 1985; Balmer and Wharton, 1989; Adlerberth et al., 2006; Jiménez et al., 2008a). Potential beneficial roles of CNS and mitis/salivarius streptococci include the competitive exclusion of undesired pathogens, such as *Staphylococcus aureus* (Kirjavainen et al., 2001; Uehara et al., 2001; Otto, 2009; Iwase et al., 2010; Park et al., 2011). The same may be applied to *Propionibacterium acnes*, another species that is commonly found in human milk. This species can prevent growth of *S. aureus* through glycerol fermentation (Shu et al., 2013) and, precisely, there is a high abundance of glycerol in human milk.

As a consequence, some human milk staphylococci, streptococci and propionibacteria may play important empirical health-promoting roles in the breastfed infant. It must be highlighted that some CNS and viridans streptococcal strains may behave as opportunistic pathogens and, therefore, a strain-by-strain rigorous safety assessment of such microorganisms must be performed before they can be intentionally administered to infants (Sanders et al., 2010; Rodríguez, 2015).

In addition, culture-independent studies have detected the presence of DNA from strict anaerobic species generally related to the digestive tract environment (*Bifidobacterium*, *Blautia*, *Faecalibacterium*, *Roseburia*, *Clostridium*, *Collinsella*, *Veillonella, Bacteroides*, *Parabacteroides*, etc.) in human milk (Rodríguez, 2014) and, also, sharing of DNA from such bacteria between maternal feces, milk and infant feces (Martín et al., 2009; Jost et al., 2013). At present, routine cultivation and commercial production of such microorganisms is not technically and economically feasible; however, advances in culturomics, functional genomics and fermentation technologies may enable the use of such microorganisms as probiotics in the future, especially considering that some of them (such as *Faecalibacterium prausnitzii*) have been associated to clear benefits to human health (Miquel et al., 2014; Martín et al., 2017). In the future, analysis of the genomes of human milk strains may contribute to a faster selection of human milk-derived bacteria in a much more rational way, and will provide additional clues on the safety, probiotic and technological properties of human milk microbes (Langa et al., 2012; Cárdenas et al., 2015).

## Modulation of the Milk Microbiota in Mothers of Preterm Infants?

There are some situations in which the composition of the milk microbiome may be improved, particularly when OMM is being administered to preterm neonates. The first one is the presence of high risk clones in milk, including methicillin-resistant and methicillin-sensitive *S. aureus*. Their presence has become increasingly common in hospitals (including maternities and NICUs) where mothers can asymptomatically acquire and transfer them to her milk and, occasionally, can lead to severe infant outcomes (Gastelum et al., 2005; Kayıran et al., 2014). However, testing OMM for high risk clones may not be a practical measure at present having in account the very low number of reports describing adverse outcomes due to the transfer of such microbes through OMM.

The second one is the antepartum, intrapartum and/or postpartum administration of antibiotics to the mother, which is relatively common among women having preterm deliveries and that, in fact, may be linked to the presence of antibiotic-resistant high risk clones. Use of antibiotics during pregnancy or lactation has been associated to a significant decrease in the number of lactobacilli- or bifidobacteria-positive human milk samples (Soto et al., 2014); these drugs are well known drivers of dysbiosis in a variety of human locations and this may lead to detrimental consequences for infant health when administered to pregnant or lactating women (Murk et al., 2011; Willing et al., 2011; Stensballe et al., 2013). A decrease in the lactic acid bacteria and bifidobacterial populations of human milk may delay colonization of the infant gut by these bacteria, making infants more susceptible to some conditions, such as allergy (Arvola et al., 2006) or colic (de Weerth et al., 2013). In contrast, a *Lactobacillus* strain isolated from human milk origin is a useful tool for the treatment of infantile colic (Sung et al., 2012).

Loss of milk lactobacilli and bifidobacteria (e.g., as a consequence of an antibiotic treatment) is also associated to negative consequences for mammary health since it uses to coincide with an overgrowth of opportunistic bacteria that, eventually, can cause mastitis (Delgado et al., 2009, 2011; Contreras and Rodríguez, 2011). Mastitis can be an additional barrier complicating the collection of OMM by mothers of preterm neonates, and often leading to an undesired abandon of lactation. Recent studies have shown that some lactobacilli strains isolated from human milk can be applied to treat or prevent mastitis (Arroyo et al., 2010; Fernández et al., 2014, 2016; Vázquez-Fresno et al., 2014; Maldonado-Lobón et al., 2015a; Espinosa-Martos et al., 2016; Hurtado et al., 2017), highlighting their potential roles in mammary homeostasis.

Use of antibiotics, chemotherapy and acute and subacute mastitis are characterized by a decreased bacterial diversity in human milk (Delgado et al., 2008; Urbaniak et al., 2014; Jiménez et al., 2015; Patel et al., 2017). The impact of this decreased bacterial diversity for infant health are unknown but previous studies suggest that it may have negative consequences at the short and long term (Mshvildadze et al., 2010; Giongo et al., 2011; Madan et al., 2012a,b).

## Human Milk-Like Minimal or Synthetic Microbiotas: The Perfect Probiotic for Preterm Infants?

The concept of “*minimal/synthetic microbiota*” derives from the fecal transplant practice (de Vos, 2013). It consists in the administration of a fecal suspension obtained from a healthy donor to a diseased person through gastroduodenoscopy, colonoscopy or enema. As a consequence, the intestinal microbiota is transferred from one person to another, including not only the cultivable microorganisms but also those that cannot currently be cultivated and which, consequently, cannot be administered in the form of a conventional probiotic. This increasingly popular practice has shown a high efficacy in the treatment of recurrent pseudomembranous colitis by *C. Difficile* (Bakken et al., 2011; Gough et al., 2011; van Nood et al., 2013).

However, fecal transplantation faces important practical challenges, including the potential transfer of undetected pathogenic viruses or harmful metabolites, or the difficulty or impossibility for a industrial standardization. Therefore, there is a need to design and develop new biotechnological processes that allow applying the principle of fecal transfer in a reproducible way. In this sense, the concept of minimal or synthetic niche-specific microbial communities can open new therapeutic ways to modify the microbiota of people with different pathologies and overcomes many of the limitations of fecal transfer (Allen-Vercoe et al., 2012; de Vos, 2013). Although this approach also has some challenges for routine practical application, a pioneering study revealed the ability of a minimal microbiota designed in the laboratory to cure recurrent *C. difficile* infection (Petrof et al., 2013). The development of human milk-based defined minimal microbiotas seems an appealing novel strategy for the premature population in order to decrease NEC and sepsis rates in the context of the emergence of multiresistant pathogenic bacteria and a higher preterm viability at lower gestational ages.

Similarly to personalization of DHM with OMM, a synthetic human milk microbiota may contain both QPS and non-QPS species, which may represent -at least at a medium term- a relevant issue for ethical approval. Initially human milk-derived consortia including only strains belonging to QPS species (mainly lactic acid bacteria and bifidobacteria) may be designed, developed and, eventually, tried. Human milk usually contains a high diversity of bacterial strains but at a rather low concentration; this is in contrast with probiotic-supplemented infant formula, which often contain one or two strains but a notably higher concentration. Many studies will be necessary to define a cultivable core human milk microbiota and the functions that the components of a synthetic microbiome should play in premature babies, which may range from prevention of infectious diseases to neuromodulation. The dosis of each strain has to be carefully considered: should each strain be present at the same concentration or their relative proportion in human milk should be respected? Developing human milk-like minimal microbiotas suited for preterm neonates is a major challenge in the frontiers of our current knowledge on the human microbiota.

## Author Contributions

All the authors participated in the critical analysis of the literature in the field, wrote and revised the manuscript.

## Conflict of Interest Statement

The authors declare that the research was conducted in the absence of any commercial or financial relationships that could be construed as a potential conflict of interest.
